# LukS‐PV induces apoptosis in acute myeloid leukemia cells mediated by C5a receptor

**DOI:** 10.1002/cam4.2137

**Published:** 2019-04-06

**Authors:** Peng Zhang, Wen‐Wei Yu, Jing Peng, Liang‐Fei Xu, Chang‐Cheng Zhao, Wen‐Jiao Chang, Xiao‐Ling Ma

**Affiliations:** ^1^ School of Medicine Shandong University Jinan Shandong China; ^2^ Department of Clinical Laboratory Anhui Provincial Hospital Hefei Anhui China; ^3^ Department of Clinical Laboratory First Affiliated Hospital of Anhui Medical University Hefei Anhui China; ^4^ Department of Clinical Laboratory Anhui Provincial Hospital of Infectious Disease Hefei Anhui China; ^5^ Department of Clinical Laboratory, The First Affiliated Hospital of USTC Division of Life Sciences and Medicine University of Science and Technology of China Hefei Anhui China

**Keywords:** AML, C5a receptor, LukS‐PV, molecular marker, therapeutic target

## Abstract

LukS‐PV is one of the two components of Panton‐Valentine leucocidin (PVL). Our previous study showed that LukS‐PV can induce apoptosis in human acute myeloid leukemia (AML) THP‐1 and HL‐60 cells. C5aR (C5a receptor) is the receptor for PVL, but whether C5aR plays a key role in LukS‐PV induced apoptosis is unclear. The aim of this study was to establish whether C5aR plays a physiological role in apoptosis of leukemia cells induced by LukS‐PV. We investigated the role of C5aR in leukemia cell apoptosis induced by LukS‐PV by pretreatment of THP‐1 and HL‐60 cells with C5aR antagonist and transfection to knockdown C5aR in THP‐1 cells or overexpress C5aR in Jurkat cells before treatment with LukS‐PV. Cell apoptosis was analyzed by staining with Annexin V/propidium iodide or Annexin V‐PE/7‐AAD. Mitochondrial membrane potential (MMP) was determined using JC‐1 dye. The expression of apoptosis‐associated genes and proteins was identified by qRT‐polymerase chain reaction and Western blotting analysis, respectively. As the C5aR antagonist concentration increased, the rate of apoptosis induced by LukS‐PV decreased, the MMP increased, and expression of pro‐apoptotic Bax and Bak genes and proteins was downregulated while that of anti‐apoptotic Bcl‐2 and Bcl‐x genes and proteins was upregulated. Knockdown of C5aR also decreased LukS‐PV–induced THP‐1 cell apoptosis. LukS‐PV did not induce apoptosis of Jurkat cells, which have no endogenous C5aR expression; however, LukS‐PV did induce apoptosis in Jurkat cells after overexpression of C5aR. Correspondingly, the MMP decreased and Bax and Bak were upregulated while Bcl‐2 and Bcl‐x were downregulated. LukS‐PV can induce apoptosis in AML cells by targeting C5aR. C5aR may be a potential therapeutic target for AML and LukS‐PV is a candidate targeted drug for the treatment of AML.

## 1 INTRODUCTION

Leukemia is a common malignancy in humans and especially in children,^1^ representing approximately one‐third of pediatric cancers.^2^ Leukemia is characterized by the inhibition of apoptosis and acceleration of proliferation, resulting in unlimited growth of immature cells.^3^ Approximately 3 800 children are diagnosed with acute myeloid leukemia (AML) annually in the United States.^4^ Treatments for AML include chemotherapy, radiotherapy, steroids, growth factors, and stem cell transplants.^5^ Despite advances in AML treatment, the 5‐year survival rate remains low.^6^ Alternative technologies that improve or replace traditional methods are being developed for the treatment of leukemia.

Bacterial toxins are a promising group of bioactive compounds and potential anticancer drugs, and some bacterial toxins have been used in cancer treatment. Botulinum neurotoxin type A, produced by strains of *Clostridium botulinum, *exhibits anticancer activity in prostate cancer and breast cancer^7^. Diphtheria toxin obtained from *Corynebacterium diphtheriae,* exhibits anticancer activity in various preclinical models, including adrenocortical carcinoma, glioblastoma, cutaneous T cell lymphoma, breast carcinoma, and cervical adenocarcinoma.^8^ Exotoxin A, secreted by *Pseudomonas aeruginosa, *exhibits anticancer activity in pancreatic cancer, melanoma, head and neck squamous carcinoma, Burkitt's lymphoma, and leukemia.^9^ Listeriolysin, produced by strains of *Listeria* monocytogenes, exhibits anticancer activity in breast carcinoma and leukemia.^10^

Panton‐Valentine leukocidin (PVL), composed of subunits LukS‐PV and LukF‐PV, is a toxin produced by *Staphylococcus aureus. *Data suggest that PVL can induce neutrophil death in vitro, characterized by apoptosis at low concentrations and necrosis at high concentrations.^11^, ^12^ We previously showed that the LukS‐PV subunit of PVL is able to induce apoptosis and cell cycle arrest in AML cells (THP‐1 cells) without membrane perforation 13.

It is generally accepted that C5aR is a receptor for PVL^14^. Thus, we speculated that LukS‐PV induces apoptosis by targeting C5aR. In this study, we used C5aR antagonist and lentivirus‐mediated silencing and overexpression of C5aR to explore the relationship between C5aR expression and apoptosis induced by LukS‐PV.

## 2 MATERIALS AND METHODS

### 2.1 Cell culture

The human AML cell lines THP‐1 and HL‐60 and the ALL cell line Jurkat were purchased from the Shanghai Institutes for Biological Sciences at the Chinese Academy of Science (Shanghai, China). Cells were cultured in RPMI‐1640 (Gibco, Grand Island, NY) supplemented with 10% (v/v) fetal bovine serum (Gibco, Carlsbad, CA) and 1% (v/v) penicillin/streptomycin in an incubator under a humidified atmosphere of 5% CO_2_ at 37°C. Media change was performed every 2‐3 days to maintain cell growth.

### 2.2 Production and purification of recombinant LukS‐PV

The LukS‐PV sequence was amplified from PVL‐positive *S. aureus* isolates by polymerase chain reaction (PCR) using the following primers: LukS‐PV, 5′‐acgcGGATCCGAATCTAAAGCTGATAACAATATTGAGAATATTG‐3′; LukS‐RV, 5′‐accgCTCGAGTCAATTATGTCCTTTCACTTTAATTTCATGAG‐3′.^15^ The PCR products were digested with XhoI and BamHI (Promega, Madison, WI) and ligated into the pET28a vector (Roche Diagnostics Corp., Basel, Switzerland) to produce six recombinant His‐tagged LukS‐PV proteins. Purification of recombinant LukS‐PV was performed as described previously.^12^

### 2.3 C5aR antagonist and lentivirus‐mediated silencing and overexpression of C5aR

The lentiviral vector used to silence and overexpress C5aR (Genechem Corp., Shanghai, China) was added to THP‐1 (C5aRsi) and Jurkat (C5aRo) cells at a multiplicity of infection of 50 prior to LukS‐PV treatment. Negative control viruses were hU6‐MCS‐Ubiquitin‐EGFP‐IRES‐puromycin for THP‐1 and Ubi‐MCS‐3FLAG‐SV40‐EGFP‐IRES‐puromycin for Jurkat. Control cells were treated with phosphate‐buffered saline. After 1 week of transfection, the expression of green fluorescent protein (GFP) and C5aR were examined by fluorescence microscopy (Nikon, Tokyo, Japan) and flow cytometry (FCM, BD Biosciences, Franklin Lakes, NJ), respectively. A C5aR antagonist, PMX53, which was dissolved in acetonitrile and added to THP‐1 and HL‐60 cells at doses of 50 nmol/L, 100 nmol/L, or 150 nmol/L.

### 2.4 Cell pretreatment

THP‐1 cells were transfected with a lentiviral vector to silence C5aR and Jurkat cells were transfected with a lentiviral vector overexpressing C5aR before culture in the absence or presence of LukS‐PV (1.0 µmol/L). PMX53 was added to the culture medium of THP‐1 and HL‐60 cells at various concentrations (50, 100, and 150 nmol/L) before treatment with LukS‐PV.

### 2.5 Cell apoptosis

All cell treatment groups were incubated in 6‐well plates at a density of 1 × 10^6^ cells per well in a humidified atmosphere of 5% CO_2_ at 37°C for 24 hours. Apoptosis was quantified using an Annexin V‐PE/7‐AAD staining kit (Trevigen, Gaithersburg, MD) for THP‐1 and Jurkat cells transfected with lentiviral vector or an Annexin V/PI staining kit (Bender Medsystems Corp., Shanghai, China) for THP‐1 and HL‐60 cells treated with PMX53. THP‐1 and Jurkat cells transfected with lentiviral vector were resuspended in a buffer (10 mM/L HEPES [pH 7.4], 140 mM/L NaCl, and 2.5 mM/L CaCl_2_) containing APC Annexin V‐PE and 7‐AAD. The cells were gently vortexed and incubated for 15 minutes at 25°C in the dark. THP‐1 and HL‐60 cells treated with PMX53 were harvested, incubated in a buffer (10 mM/L HEPES/NaOH [pH 7.4], 140 mM/L NaCl, and 2.5 mM/L CaCl_2_) containing Annexin V‐EGFP (1 Ag/mL), and resuspended in HEPES buffer containing 0.25 Ag/mL propidium iodide. The cells were gently vortexed and incubated for 15 minutes at 25°C in the dark. Apoptosis was quantified by flow cytometry (FCM) within 1 hour. All experiments were performed three times. The data were analyzed using FCS Express software Flowjo 7.6.

### 2.6 Mitochondrial membrane potential

After culturing in the absence or presence of LukS‐PV, THP‐1, HL‐60, and Jurkat cells were washed twice with phosphate‐buffered saline and resuspended in JC‐1 staining solution at 37°C for 15 minutes in a 5% CO_2_ incubator. The cells were centrifuged and resuspended in 500 µL of incubation buffer preheated to 37°C. MMP was analyzed by FCM within 1 hour and cells were observed by fluorescence microscopy. All experiments were performed three times. The data were analyzed by FCS Express software.

### 2.7 Reverse‐transcription PCR analysis

RNA was extracted from THP‐1, HL‐60, and Jurkat cells and examined by UV spectrophotometry based on the optical density ratios at 260 nm and 280 nm. The absorbance ratio was 1.8‐2.1. Total RNA was reverse transcribed using the QuantiNova SYBR Green PCR kit (QiaGen GmbH, Hilden, Germany) on an ABI7500 real‐time PCR system (Applied Biosystems, Foster City, CA). Relative mRNA expression levels of apoptosis‐related genes were determined using the following gene‐specific primers: *BAK*: 5′‐ATGTCGTCCCCGATGATG‐3′ and 5′‐AGGAACAGGAGGCTGAAGG‐3′; *BAX*: 5′‐CCGATTCATCTACCCTGCTG‐3′ and 5′‐TGAGCAATTCCAGAGGCAGT‐3′; *BCL‐2*:5′‐GAGACAGCCAGGAGAAATCAA‐3′ and 5′‐ATGTGTGTGGAGAGCGTCAA‐3′; *BCL‐X*: 5′‐GGTCTCCATCTCCGATTCAGT‐3′ and 5′‐CTGGTGGTTGACTTTCTCTCCT‐3′. Statistical analyses were performed using the ΔΔCt values and *GAPDH* served as an internal control. The experiment was repeated three times.

### 2.8 Western blotting analysis

A nuclear extraction kit (Epigentek, Brooklyn, NY) was used to separate the cytosolic and nuclear fractions of total proteins from THP‐1, HL‐60, and Jurkat cells according to the manufacturer's instructions. Semi‐quantitative immunoblot data were generated using Scion imaging software (scion‐image.software.informer.com). Proteins were separated on a 12% SDS‐PAGE gel and transferred to a nitrocellulose membrane. The membrane was incubated overnight at 4°C with anti‐BAX (1:800), anti‐BCL‐2 (1:800), and anti‐GAPDH (1:1000) primary antibodies (Cell Signaling Technology, Beverly, MA). The membrane was incubated with an appropriate secondary antibody conjugated to horseradish peroxidase for 1 hour. The complexes were visualized using ECL reagent (Pierce, Rockford, IL). The experiment was repeated three times.

### 2.9 Statistical analysis

All data are represented as the means ± standard deviation. Significant differences between groups were determined through one‐way ANOVA using SPSS Statistics 17.0 (SPSS Inc, Chicago, IL). A value of *P* < 0.05 was considered statistically significant.

## 3 RESULTS

### 3.1 C5aR antagonist inhibited apoptosis induced by LukS‐PV in THP‐1 and HL‐60 cells

**Figure 1:**
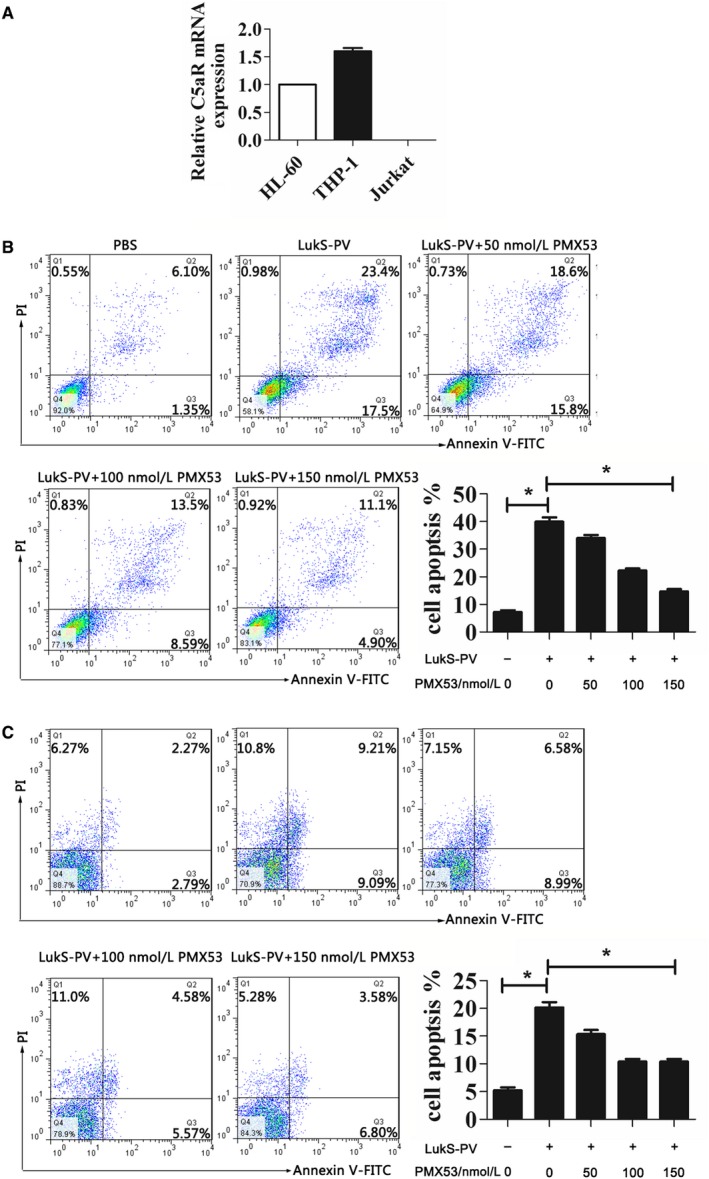
C5a receptor (C5aR) antagonist inhibited apoptosis of THP‐1 cells and HL‐60 cells induced by LukS‐PV. (A) Polymerase chain reaction (PCR) analysis of C5aR expression in leukemia cell lines. THP‐1 and HL‐60 cells were pretreated with C5aR antagonist at the indicated concentrations prior to incubation with 1.0 µmol/L LukS‐PV for 24 hours. (B, C) Flow cytometry using AnnexinV‐FITC staining shows that C5aR antagonist decreased LukS‐PV–induced apoptosis of THP‐1 (B) and HL‐60 (C) cells

Our previous study showed that LukS‐PV has the ability to induce apoptosis in AML THP‐1 and HL‐60 cells, to investigate the role of C5aR in leukemia cell apoptosis induced by LukS‐PV, we first measured endogenous C5aR protein levels in three human leukemia cell lines (HL‐60, THP‐1, and Jurkat). Real‐time PCR analysis showed that mean C5aR expression was highest in THP‐1 cells, lower in HL‐60, and almost undetectable in Jurkat cells (Figure 1A).

**Figure 2:**
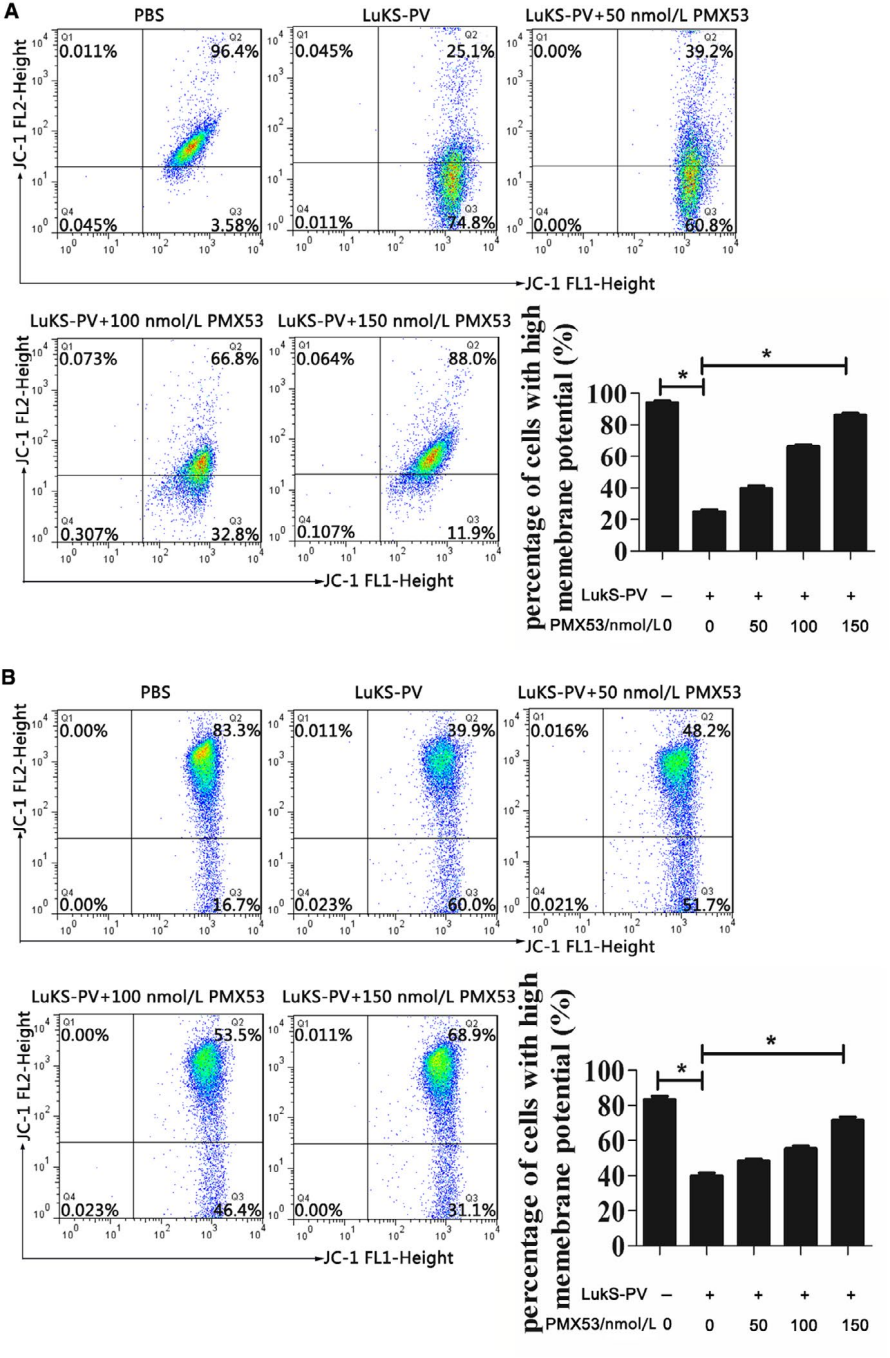
Effects of C5a receptor (C5aR) antagonist on mitochondrial membrane potential (MMP) in THP‐1 and HL‐60 cells after LukS‐PV treatment. THP‐1 and HL‐60 cells were exposed to 1.0 µmol/L LukS‐PV for 24 hours after pretreatment with the indicated concentrations of antagonist. JC‐1 staining shows that C5aR antagonist increased the MMP in THP‐1 (A) and HL‐60 (B) cells

**Figure 3:**
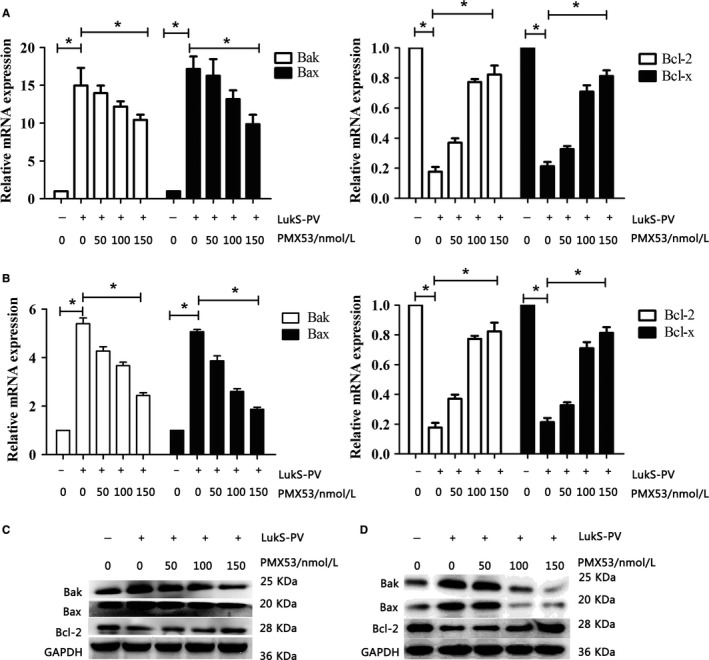
Effects of C5a receptor (C5aR) antagonist on apoptosis‐associated genes and proteins in THP‐1 and HL‐60 after LukS‐PV treatment. THP‐1 and HL‐60 cells were treated with 1.0 µmol/L LukS‐PV for 24 hours after pretreatment with 50, 100, or 150 nmol/L PMX53 and the expression of apoptosis‐associated genes and proteins in THP‐1 (A, C) and HL‐60 (B, D) cells was identified by qRT‐polymerase chain reaction (PCR) and Western blotting analysis

We next studied the effect of the C5aR antagonist PMX53 on LukS‐PV–induced apoptosis. THP‐1 cells and HL‐60 cells were pretreated with different concentrations of PMX53 and then treated with LukS‐PV. Compared with the PBS control group, the LukS‐PV treatment groups showed significantly increased apoptosis of THP‐1 and HL‐60 cells (Figure 1B,C). As the concentration of C5aR antagonist increased, the rate of apoptosis decreased from 39.97% ± 2.72% to 14.67% ± 1.53% in THP‐1 cells (Figure 1B) and from 20.7% ± 1.67% to 10.38% ± 1.02% in HL‐60 cells (Figure 1C). Concurrently, the MMP increased from 25.07% ± 2.25% to 86.33% ± 2.08% in THP‐1 cells (Figure 2A) and from 39.87% ± 3.05% to 71.53% ± 3.27% in HL‐60 cells (Figure 2B), and expression of the pro‐apoptotic Bax and Bak genes and proteins was downregulated while that of the anti‐apoptotic Bcl‐2 and Bcl‐x genes and proteins was upregulated (Figure 3A‐D). These results showed that C5aR antagonist notably dampened LukS‐PV–induced apoptosis of leukemic cells.

### 3.2 Knockdown of C5aR expression inhibited apoptosis of THP‐1 cells induced by LukS‐PV

**Figure 4:**
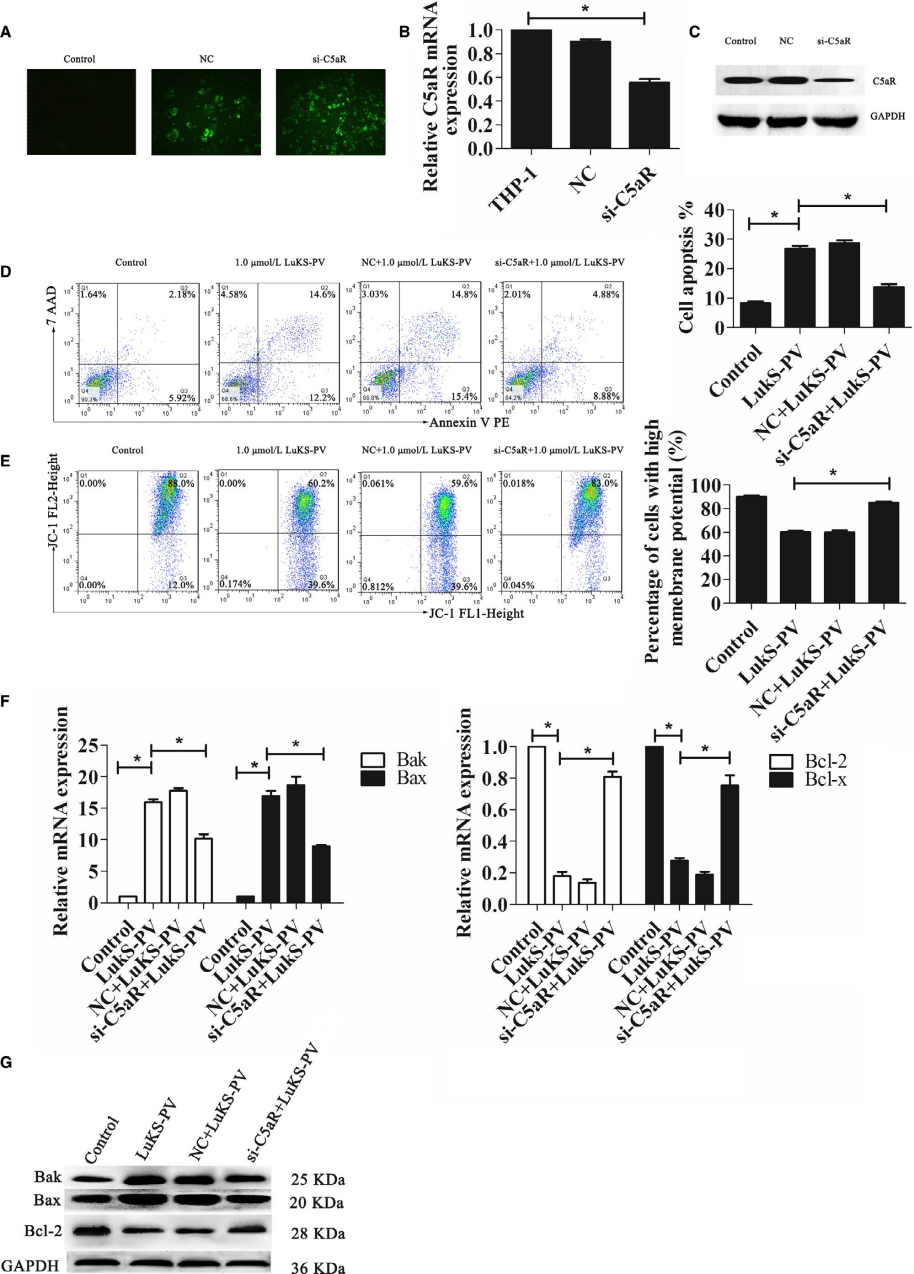
Knockdown of C5a receptor (C5aR) expression inhibits apoptosis of THP‐1 induced by LukS‐PV. (A) Green fluorescent protein (GFP)‐labeled lentivirus in THP‐1 cells was observed by fluorescent microscopy. (B) Relative expression of C5aR mRNA in indicated cell groups measured by qRT‐polymerase chain reaction (PCR). (C) Expression of C5aR protein in indicated cell groups measured by Western blotting. (D) THP‐1 cells treated with control siRNA or C5aR siRNA were exposed to 1.0 µmol/L LukS‐PV for 24 hours. Flow cytometric analysis of Annexin V‐PE/7‐AAD staining shows that the knockdown of C5aR inhibited apoptosis induced by LukS‐PV. (E) Flow cytometric analysis of JC‐1 staining shows increased mitochondrial membrane potential (MMP) in si‐C5aR THP‐1 cells. (F, G) The expression of apoptosis‐associated genes and proteins was identified by qRT‐PCR (F) and Western blotting analysis (G)

To further research the role of C5aR in leukemia cell apoptosis induced by LukS‐PV, we transfected THP‐1 cells with siRNA to silence C5aR expression and analyzed green fluorescence in the cells by fluorescence microscopy after 1 week (Figure 4A). The levels of C5aR RNA and protein in THP‐1 cells were confirmed by RT‐PCR (Figure 4B) and Western blotting (Figure 4C).

THP‐1 and C5aR‐silenced (si‐C5aR) THP‐1 cells were treated with LukS‐PV as indicated in Figure 4D‐G. Compared with the control group, LukS‐PV significantly increased apoptosis in THP‐1 cells, but this effect was markedly alleviated in si‐C5aR THP‐1 cells; compared with non‐silenced cells, the rate of apoptosis decreased from 28.6% ± 1.76% to 13.69% ± 1.75% (Figure 4D), the MMP increased from 59.87% ± 3.01% to 85% ± 2.1% (Figure 4E), the pro‐apoptotic genes Bax and Bak were downregulated, and the anti‐apoptotic genes Bcl‐2 and Bcl‐x were upregulated (Figure 4F,G).

### 3.3 Overexpression of C5aR increased apoptosis of Jurkat cells induced by LukS‐PV

**Figure 5:**
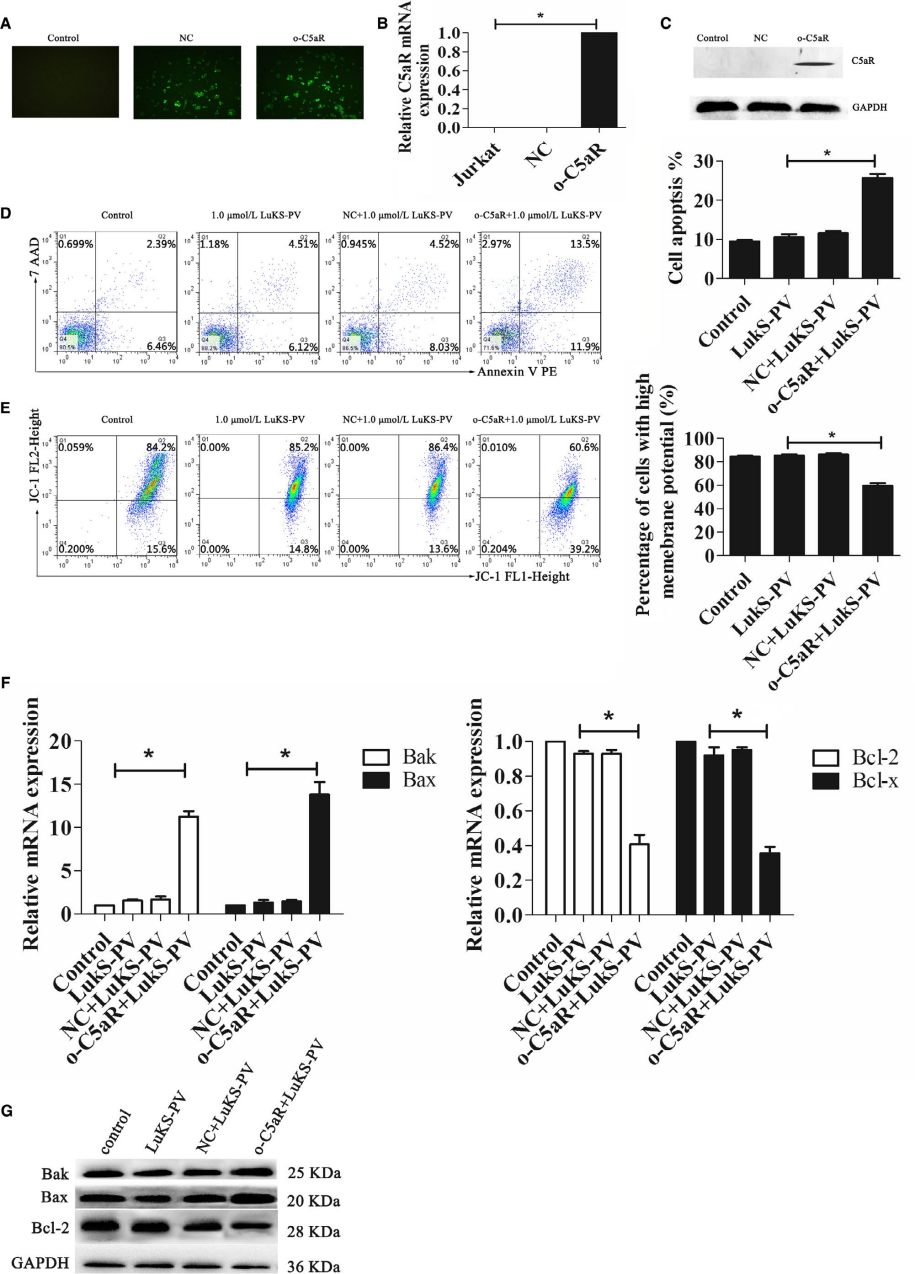
Overexpression of C5a receptor (C5aR) in Jurkat cells increased LukS‐PV–induced apoptosis. (A) Green fluorescent protein (GFP)‐labeled lentivirus in Jurkat cells was observed by fluorescent microscopy. (B) Relative expression of C5aR mRNA in indicated cell groups measured by qRT‐polymerase chain reaction (PCR). (C) Expression of C5aR protein in indicated cell groups measured by Western blotting. (D) Jurkat cells treated with a control siRNA or C5aR‐overexpressing siRNA were exposed to 1.0 µmol/L LukS‐PV for 24 hours. Flow cytometric analysis of Annexin V‐PE/7‐AAD staining shows that overexpression of C5aR increased apoptosis of Jurkat cells induced by LukS‐PV. (E) Flow cytometric analysis of JC‐1 staining shows decreased mitochondrial membrane potential (MMP) in o‐C5aR Jurkat cells. (F, G) The expression of apoptosis‐associated genes and proteins was identified by qRT‐PCR (F) and Western blotting analysis (G)

We also overexpressed C5aR expression in Jurkat cells, which have no endogenous C5aR expression. Successful transfection was confirmed by fluorescence microscopy (Figure 4A), RT‐PCR (Figure 5B), and Western blotting (Figure 5C).

Jurkat and C5aR‐overexpressing (o‐C5aR) Jurkat cells were treated with LukS‐PV for 24 hours. As shown in Figure 5D, the apoptosis rates of the control group and the LukS‐PV treatment group were 9.48% ± 0.69% and 10.58% ± 1.25% and the MMP was 84.3% ± 1.65% and 85.4% ± 1.51%, respectively (Figure 5E), and these differences were not statistically significant (*P* > 0.05). In addition, the expression of apoptosis‐associated genes and proteins were not significantly changed (Figure 5F,G). These results indicated that LukS‐PV did not induce apoptosis of Jurkat cells lacking endogenous C5aR expression. However, in LukS‐PV–treated Jurkat cells that overexpressed C5aR the rate of apoptosis increased from 10.58% ± 1.25% to 25.7% ± 1.77% (Figure 5D), the MMP decreased from 85.4% ± 1.51% to 59.53% ± 4.11% (Figure 5E), and the pro‐apoptotic Bax and Bak genes and proteins were upregulated while the antiapoptotic Bcl‐2 and Bcl‐x genes and proteins were downregulated (Figure 5F,G), compared with Jurkat cells.

## 4 DISCUSSION

PVL is composed of two subunits, LukS‐PV and LukF‐PV. Our previous study showed that LukS‐PV can induce apoptosis and inhibit proliferation in AML THP‐1 and HL‐60 cells, and in vivo studies have shown that LukS‐PV has no obvious side effects.^15^ C5aR is a receptor for PVL, LukS‐PV specifically binds to receptors on the plasma membrane of polymorphonuclear leukocytes and then combines with LukF‐PV to form an octamer that plays a key role in membrane perforation. However, whether binding of LukS‐PV to C5aR is required for the induction of apoptosis is unclear. Here, we studied the effects of C5aR on LukS‐PV–induced apoptosis by three intervention approaches: treatment with C5aR antagonist, knockdown of C5aR, and overexpression of C5aR. We found that C5aR antagonist and silencing C5aR suppressed the induction of apoptosis by LukS‐PV, whereas overexpression of C5aR increased apoptosis, indicating that C5aR is a target for LukS‐PV to induce apoptosis in leukemia cells.

Apoptosis involves three main signal transduction pathways: the mitochondrial pathway, death receptor pathway, and endoplasmic reticulum stress pathway. In our initial experiments using gene chip detection we found that LukS‐PV upregulated the expression of Bax, caspase3, caspase8, and caspase9 and downregulated the expression of Bcl‐2. This indicates that LukS‐PV induces THP‐1 apoptosis predominantly through the mitochondrial pathway. Here, we examined mitochondrial pathway‐associated markers and found that LukS‐PV induces apoptosis at a much lower level in cells pretreated with the C5aR antagonist PMX53 or with knockdown of C5aR, compared with the LukS‐PV treated control group, together with a higher MMP, downregulated expression of pro‐apoptotic genes and proteins (Bax/Bak), and upregulated expression of anti‐apoptotic genes and proteins (Bcl‐2). Overexpression of C5aR had the opposite effects. Together, these changes demonstrate that LukS‐PV induces leukemia cell apoptosis via the mitochondrial signal transduction pathway by targeting C5aR.

C5aR (also known as CD88) is a G‐protein–coupled receptor encoded by a gene on chromosome 19q13.3‐19q13.4.^16^ C5aR was originally reported to be expressed only in myeloid cells with no expression in lymphocytes and red blood cells, but a recent study found that many non‐myeloid–derived tumor cells also express C5aR, including lung cancer^17^, liver cancer,^18^ gastric cancer,^19^ renal cancer,^20^ breast cancer,^21^ and colon cancer^22^, and that the expression of C5aR is significantly higher in tumor cells compared with non‐tumorous tissues. Moreover, high expression of C5aR is associated with cancer progression, distant metastasis, and poorer outcome. The above findings indicate that C5aR plays an important role in tumor progression. Our study showed that LukS‐PV–induced apoptosis by targeting C5aR, and we speculate that LukS‐PV may have therapeutic effects on solid tumors with high expression of C5aR.

In conclusion, LukS‐PV can induce apoptosis in AML cells through C5aR, implicating C5aR as a potential target and LukS‐PV as a targeted drug for the treatment of AML.

## CONFLICTS OF INTEREST

There are no conflicts of interest for any author.
